# Evaluation of the Performance of the Loopamp Trypanosoma cruzi Detection Kit for the Diagnosis of Chagas Disease in an Area Where It Is Not Endemic, Spain

**DOI:** 10.1128/JCM.01860-20

**Published:** 2021-04-20

**Authors:** Maria D. Flores-Chavez, Alba Abras, Cristina Ballart, Ismael Ibáñez Perez, Pilar Perez-Gordillo, Montserrat Gállego, Carmen Muñoz, Zaira Moure, Elena Sulleiro Igual, Javier Nieto, Emilia García Diez, Israel Cruz, Albert Picado

**Affiliations:** a National Centre for Microbiology, Instituto de Salud Carlos III, Madrid, Spain; b Fundación Mundo Sano-España, Madrid, Spain; c Secció de Parasitologia, Departament de Biologia, Sanitat i Medi Ambient, Facultat de Farmàcia i Ciències de l’Alimentació, Universitat de Barcelona, Barcelona, Spain; d Departament de Biologia, Universitat de Girona, Girona, Spain; e ISGlobal, Hospital Clínic–Universitat de Barcelona, Barcelona, Spain; f Servei de Microbiologia, Hospital de la Santa Creu i Sant Pau, Barcelona, Spain; g Departament de Genètica i Microbiologia, Universitat Autònoma de Barcelona, Cerdanyola del Vallès, Spain; h Hospital de la Vall d’Hebron, Barcelona, Spain; i National School of Public Health, Instituto de Salud Carlos III, Madrid, Spain; j Foundation for Innovative New Diagnostics FIND, Geneva, Switzerland; Mayo Clinic

**Keywords:** congenital Chagas disease, LAMP, molecular diagnosis, sensitivity, specificity, accuracy

## Abstract

In Spain, PCR is the tool of choice for the diagnosis of congenital Chagas disease (CD) and serology for diagnosing chronic CD. A loop-mediated isothermal amplification test for Trypanosoma cruzi DNA detection showed good analytical performance and ease of use. We aimed to evaluate the performance of the Loopamp Trypanosoma cruzi detection kit (Eiken Chemical Co. Ltd., Japan) (*Tcruzi*-LAMP) for congenital and chronic CD diagnosis using well-characterized samples. We included samples from 39 congenital and 174 chronic CD cases and from 48 uninfected children born to infected mothers and 34 nonchagasic individuals. The sensitivity, specificity, and accuracy of *Tcruzi*-LAMP were estimated using standard case definitions for congenital CD (positive result by parasitological or PCR tests or serology after 9 months of age) and chronic CD (positive serology by at least two tests). The *Tcruzi*-LAMP results were read by visual examination and a real-time fluorimeter. For congenital CD, *Tcruzi*-LAMP sensitivity was 97% for both types of reading; specificity was 92% by visual examination and 94% by fluorimeter. For chronic CD, sensitivity was 47% and specificity 100%. The accuracy in congenital CD was >94% versus 56% in chronic CD. The agreement of *Tcruzi*-LAMP with PCR tests was better in congenital CD (kappa, 0.86 to 0.91) than in chronic CD (kappa, 0.67 to 0.83). The Loopamp Trypanosoma cruzi detection kit showed good performance for the diagnosis of congenital CD. *Tcruzi*-LAMP, like PCR, can be useful for the screening and early diagnosis of congenital infection.

## Abstract (precis)

In Spain, PCR is the tool of choice for the diagnosis of congenital Chagas disease (CD) and serology for diagnosing chronic CD. A loop-mediated isothermal amplification test for Trypanosoma cruzi DNA detection showed good analytical performance and ease of use.

## INTRODUCTION

In Spain, Trypanosoma cruzi infection, or Chagas disease (CD), is one of the major imported parasitic diseases (1). Although vector transmission does not occur in Spain, there are autochthonous cases due to blood transfusion, organ transplant, and congenital transmission (from mothers to children during pregnancy or delivery). Also, there are carriers of chronic CD, and it has been estimated that between 47,984 and 86,618 infected people could be residing in Spain (2). However, the number of cases diagnosed is unknown, as CD is not included in the category of notifiable diseases except in the region of Catalonia (3). Records from the National Centre for Microbiology (CNM, Spanish acronym) from 1997 to the present show more than 6,000 infected individuals, of which 51 were congenital cases, 5 were transfusion-associated cases, and 2 were due to transmission through organ transplant (CNM data, unpublished).

The serological screening of at-risk blood donors has been mandatory since 2005 (4). Also, half of the Spanish regional health authorities recommend serological screening in pregnant women and their newborns, with the regions of Catalonia, Galicia, and Valencia pioneering the inclusion of a diagnostic algorithm for congenital CD in their official guidelines (3, 5–7). In the rest of Spain, serological screening in pregnant women and at-risk migrants is also conducted in the main public hospitals at the discretion of their health professionals.

Most of the CD cases in settings of nonendemicity are in the chronic phase, when the parasite burden is usually low and intermittent (8–10). Therefore, suspected chronic CD is usually confirmed by serological tests (11). Since seroreversion to negative following successful treatment of a chronic CD case may take years, serology is still recommended for treatment follow-up (12). Although PCR is the best option for early detection of circulating parasites, in chronic patients, it is mainly used as a marker of therapeutic failure (8, 9).

In Spain, the acute phase is observed mainly in newborns and is characterized by high degree of parasitemia, making diagnosis by parasitological tests feasible (11). Unfortunately, the sensitivity of parasitological tests is suboptimal, and infants who are diagnosed by serology 9 to 10 months after birth, when maternal antibodies disappear (13–15), are often missed. In Europe, in-house or commercial PCR tests are routinely used to diagnose congenital CD cases (16–18). Real-time PCRs targeting satellite DNA (SatDNA) or kinetoplast (kDNA) minicircles are commonly used (9, 19, 20).

Years ago, Eiken Chemical Co. Ltd., Japan, developed a simple approach for T. cruzi DNA detection based on the loop-mediated isothermal amplification test (*Tcruzi-*LAMP). This test amplifies the T. cruzi SatDNA in less than 1 h, under isothermal conditions, without specific instrumentation, and interpretation of results is quite straightforward (21). Besuschio et al. reported good analytical sensitivity and specificity for this test and showed its potential usefulness in a small number of clinical samples (21). Based on this, we aimed to evaluate the sensitivity, specificity, and accuracy of *Tcruzi*-LAMP using well-characterized samples from confirmed congenital and chronic CD cases born and living in Spain. We also estimated the agreement between *Tcruzi*-LAMP and PCR tests targeting kDNA and SatDNA.

## MATERIALS AND METHODS

**Study design.** The evaluation was a retrospective study using convenience sampling from the clinical specimen collections of the CNM, Instituto de Salud Carlos III (ISCIII), Madrid, Pharmacy and Food Sciences Faculty, Universitat de Barcelona (PFSF-UB), Barcelona, and Hospital de la Santa Creu i Sant Pau (HSCSP), Barcelona. The time frame of sample and data collection was between 2003 and 2016 (Table 1), and the convenience sampling was performed considering eligibility criteria (see below).

**TABLE 1:** Source and matrix of sample series^*a*^

Group	Collection source	Yr of blood collection	Condition of storage	Matrix	No. of samples
Congenital CD cases	CNM	2008–2016	Refrigerated	GEB	29
	PFSF-UB/HSCSP	2003–2016	Frozen	DNA from EB	10
Uninfected children	CNM	2008−2016	Refrigerated	GEB	23
	PFSF-UB/HSCSP	2003–2016	Frozen	DNA from EB	25
Chronic CD cases	CNM	2014–2016	Refrigerated	GEB	174
Nonchagasic individuals	CNM	2014–2016	Refrigerated	GEB	34

a CD, Chagas disease; CNM, National Centre for Microbiology; PFSF-UB, Pharmacy and Food Sciences Faculty, Universitat de Barcelona; HSCSP, Hospital de la Santa Creu i Sant Pau; GEB, guanidine-EDTA-blood; EB, EDTA-blood.

The study was designed as a case-control model for evaluation of *Tcruzi*-LAMP in congenital infection; for each positive congenital case, we included one uninfected baby. The chronic samples were included to evaluate performance of LAMP in a low degree of parasitemias. The case-control model was planned considering kDNA-PCR results to have a similar number of samples with positive and negative results.

**Samples. (i) Eligibility criteria.** DNA and guanidine-EDTA-blood (GEB) samples were selected from the above-mentioned collections based on (i) previous results of laboratory routine diagnostic tests epidemiological and clinical background (see Data set S1 in the supplemental material) and (ii) availability of enough volume to run index (*Tcruzi*-LAMP) and PCR tests.

The epidemiological data considered related to CD were the country of birth, age, stays or trips to areas of endemicity, mother from an area of endemicity, and risk exposure to T. cruzi (blood transfusion, transplantation, laboratory accident). We also considered the following clinical data: presence of cardiac or digestive alterations or any other symptom related to CD. The affected population residing in Spain is generally asymptomatic; thus, epidemiological data had greater relevance for the inclusion of cases. Raw epidemiological and clinical data are included in Data set S1.

**(ii) Congenital CD cases.** A total of 39 samples from infected children born in Spain from women with CD were included. The diagnosis of congenital CD had been confirmed following the guidelines in Madrid and Catalonia, i.e., (i) before 9 months after birth, a positive result by parasitological tests (direct microscopy, microhematocrit, or culture) and/or PCR or (ii) after 9 months of age by positive serology, plus no travel to areas of endemicity from birth to time of diagnosis (3, 7, 22, 23).

**(iii) Uninfected children.** This group included 48 samples from babies born to T. cruzi infected mothers for whom congenital CD had been ruled out by negative parasitological and/or molecular tests before 9 months of age and negative serology from this point on (Table S1 and Data set 1 in supplemental material).

**(iv) Chronic CD cases.** We selected 174 samples from patients with chronic CD who had a positive result in at least two different serological tests (Table S2 and Data set S1).

**(v) Nonchagasic individuals.** A total of 34 samples from individuals at risk for T. cruzi infection for whom CD had been ruled out by negative serology (Table S2 and Data set S1) were included.

**(vi) Positive controls for molecular tests.** DNA was obtained from T. cruzi cultures of discrete typing units TcV and TcI using the High Pure PCR template preparation kit (Roche Diagnostics, Germany) following the tissue protocol. The DNA concentration was measured with a NanoDrop 1000 spectrophotometer (Thermo Fisher Scientific, USA). For the positive controls, the DNA concentration was adjusted to 10 and 1 parasites/ml. We assumed 100 fg DNA as the equivalent of one parasite (24). Standard curves for quantitation were made of 10-fold serial dilutions of T. cruzi TcV genomic DNA, spiking genomic DNA from uninfected individuals.

**Test methods. (i) Loopamp Trypanosoma cruzi detection kit (*Tcruzi*-LAMP, index test).** *Tcruzi*-LAMP was performed in a single test following the manufacturer’s instructions using a portable real-time fluorimeter (Genie III, OptiGene, UK).

DNA from GEB samples was purified using a High Pure PCR template preparation kit (Roche Diagnostics, Germany) following the recommendations of Ramírez et al. (25). The starting volume of GEB was 300 μl, and the volume of elution was 100 μl; 5 μl of DNA per reaction was tested. Amplification conditions were the first step of 5 min at 95°C, followed by 40 min at 65°C. Reading of the results was by visual examination immediately after the end of the amplification process; no color change was recorded as a negative result, and a color change, as a positive result. Results provided by the fluorimeter were displayed at the end of the reaction and recorded as the time to positivity in minutes and seconds (for more details, see Fig. S1).

Two operators were trained in the management of biological samples, DNA extraction, and molecular tests 2 weeks before starting the study; they did not participate in the selection of the samples, but performed the DNA extraction and *Tcruzi*-LAMP blinded to previous results.

**(ii) Reference tests.** *(a) Parasitological tests.* Parasitological confirmation was based on direct observation of trypomastigotes in a blood sample, either after a fresh examination or with a microhematocrit technique (MHT) (26). Alternatively, 100 μl of the blood sample was inoculated in Novy-MacNeal-Nicolle (NNN) culture medium supplemented with liquid medium liver infusion tryptose, 10% fetal bovine serum, and antibiotics; the supernatant of the culture was examined at 15 and 30 days postinoculation. These tests were performed at the time of sample collection.

*(b) Serological tests.* At CNM, serology for CD diagnosis was carried out using an in-house enzyme-linked immunosorbent assay (ELISA) and immunofluorescence antibody test (IFAT) (27) and Chagatest enzyme-linked immunosorbent assay (ELISA) recombinant V 4.0 (Wiener, Argentina), and at PFSF-UB/HSCSP, with in-house and recombinant ELISAs (BioELISA Chagas; Biokit, Lliçà d’Amunt, Spain) (16, 28). These tests were also run at the time of sample collection.

*(c) Conventional kDNA PCR (kDNA-PCR).* This PCR targets a variable sequence of kDNA minicircles. DNA purification was performed according to the standard CNM procedure (10). The starting volume of GEB samples was 300 μl, and DNA was dissolved in 75 μl of molecular-grade water. Samples and positive and negative controls were processed in duplicate. Amplification was routinely performed in a total volume of 75 μl, using 10 μl of DNA in each duplicate. Positive and negative controls were included in duplicate for each amplification run. PCR products of 330 bp were analyzed by electrophoresis on a 2% agarose gel and using GelRed nucleic acid gel stain (Biotum, USA) as the dye. For this study, the kDNA-PCR was rerun on all samples at the same time as *Tcruzi*-LAMP.

*(d) Satellite DNA real-time PCR (Sat-qPCR).* At CNM, the Sat-qPCR was performed according to Ramírez et al. (25) and Duffy et al. (29), and at PFSF-UB/HSCSP, according to Piron et al. (30), with slight modifications (16, 20). For this study, the Sat-qPCR was rerun on all samples from PFSF-UB/HSCSP at the same time as *Tcruzi*-LAMP.

**FIG 1:**
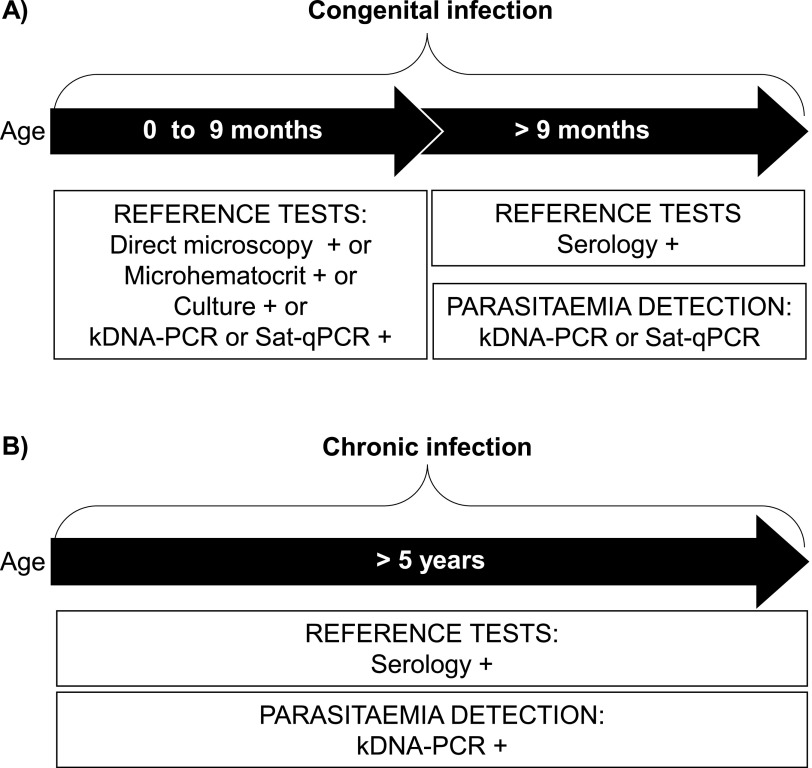
Diagram of the use and role of reference tests in the diagnosis of congenital and chronic infection of Chagas disease according to the age of the study population. (A) Children born in Spain to mothers with T. cruzi infection. (B) People born or staging in area of endemicity for T. cruzi infection living in Spain.

**Data analysis.** The composite gold standard according to age of diagnosis was used for sample classification as case or control: parasite detection by any parasitological or molecular test confirmed the T. cruzi infection before 9 months of age, after which the diagnosis was based on serology (Fig. 1). In this context, sensitivity, specificity, accuracy, and their 95% confidence interval (CI) of *Tcruzi*-LAMP were estimated by binomial distribution (http://statpages.info/confint.html). Differences regarding the composite gold standard were analyzed with McNemar’s test. kDNA-PCR or Sat-qPCR after 9 months of age are mainly used to determine the presence of parasites; thus, the agreement between molecular tests was measured with Cohen’s kappa statistic (κ) and the Spearman rank correlation (*r*_s_). The Mann-Whitney U test was used to analyze differences among time of positivity and degree of parasitemia (continuous responses) regarding the congenital and chronic CD groups (categorical factors). To explore the influence of degree of parasitemia (continuous response) in the measurement of agreement, the overall data were stratified according to the different combinations of test results for all the molecular tests. This meant that the positive cases were classified in two groups: (i) samples with positive results in all molecular tests, concordant group, and (ii) samples with a positive result in any test, discordant group (categorical factors). Differences were determined with the Mann-Whitney U test. In Sat-qPCR, not quantifiable values were considered equivalent to the limit of detection. Analyses were performed using Minitab 18.1.

**Ethical clearance.** The use of samples from different collections was approved by the ethics committees of each institution as follows: Research Ethics Committee of ISCIII, reference CEI PI17_2011; Clinical Research Ethics Committee of HSCSP, references IIBSP-CHA-2013-33 and CEIC 53/2013; and the Research Ethics Committee of Universitat de Barcelona, reference IRB00003099. All samples were anonymized for the study.

## RESULTS

**FIG 2:**
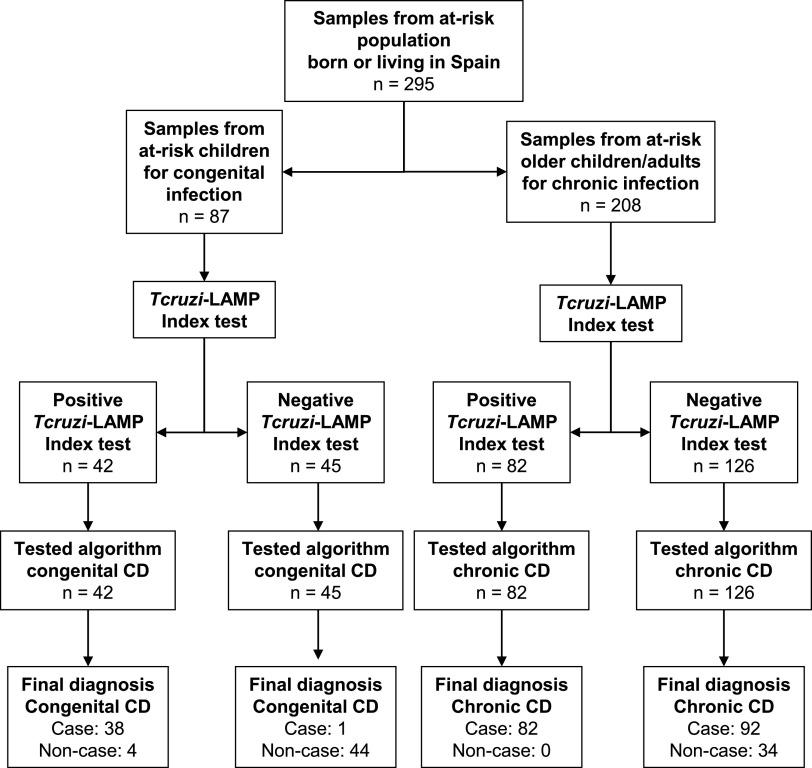
Diagram of samples and testing workflow used to evaluate the performance of *Tcruzi*-LAMP.

A total of 295 samples from an population at risk of being infected with T. cruzi were tested by *Tcruzi*-LAMP (Fig. 2).

### Congenital Chagas disease.

At CNM, parasites were seen in 21 cases out of 22 for whom a blood sample was examined by direct microscopy; for the remaining case, parasite was detected by culture (Table 2). *Tcruzi*-LAMP and PCR tests yielded positive results in all congenital CD cases. In samples from children previously testing negative for T. cruzi infection (*n* = 25), *Tcruzi*-LAMP returned a positive result in four infants when the reaction was read by visual examination, three of which were also positive by fluorimeter. All of them were negative by PCR tests.

**TABLE 2:** Characteristics of congenital CD cases^*a*^

Case	Site^*b*^	Time of sample collection after birth (days)	Symptoms compatible with CD	Par test results^*c*^	Serol test results^*e*^	kDNA-PCR results	Sat-qPCR (*C_T_*)	*Tcruzi*-LAMP	
								Visual examination	Fluorimeter^*f*^
1	1	1	No	+	+	+	22.82	+	18:45
2	1	4	No	+	+	+	33.73	+	18:00
3	1	4	No	+	+	+	22.33	+	13:00
4	1	7	Yes	+	+	+	31.98	+	28:15
5	1	10	Yes	+	+	+	19.75	+	11:45
6	1	13	No	+	+	+	14.13	+	10:45
7	1	14	No	ND	+	+	33.07	+	16:30
8	1	16	No	+	+	+	24.62	+	13:45
9	1	24	No	+^*d*^	+	+	23.08	+	16:45
10	1	25	No	+	+	+	24.11	+	17:15
11	1	31	No	+	+	+	23.08	+	14:45
12	1	35	No	+	+	+	27.54	+	12:00
13	1	35	No	+	+	+	32.44	+	13:30
14	1	42	No	+	+	+	21.57	+	16:15
15	1	49	No	+	+	+	23.35	+	11:15
16	1	53	No	+	+	+	22.02	+	13:45
17	1	61	No	+	+	+	19.95	+	13:15
18	1	74	No	+	+	+	20.72	+	14:15
19	1	83	No	+	+	+	21.03	+	14:15
20	1	93	No	+	+	+	19.92	+	16:00
21	1	115	No	+	+	+	19.04	+	11:15
22	1	238	No	+	+	+	18.50	+	13:15
23	1	480	No	ND	+	+	24.85	+	20:15
24	1	687	No	ND	+	+	29.13	+	17:00
25	1	1,081	No	ND	+	+	21.64	+	13:30
26	1	1,118	No	+	+	+	23.52	+	14:30
27	1	1,119	No	ND	+	+	24.91	+	17:45
28	1	1,425	No	ND	+	+	28.97	+	16:00
29	1	1,711	No	ND	+	+	31.74	+	16:45
30	2	1	No	ND	+	+	30.14	+	17:00
31	2	1	No	ND	+	+	12.99	+	11:15
32	2	1	No	–	+	+	26.86	+	21:45
33	2	15	Yes	–	+	+	18.77	+	12:30
34	2	30	No	ND	+	+	22.95	+	14:00
35	2	153	No	+	+	+	31.23	+	27:30
36	2	214	No	–	ND	+	24.45	+	13:15
37	2	305	No	–	ND	+	20.49	+	11:45
38	2	456	No	ND	+	+	32.18	–
39	2	730	No	–	+	+	27.31	+	16:45

a ND, not done; +, positive; –, negative; *C_T_*, cycle threshold.

b 1, CNM; 2, PFSF-UB and HSCSP.

c Par, parasitology.

d Positive by culture.

e Serol, serology.

f Time to positivity in minutes and seconds.

At PFSF-UB/HSCSP, only one case, 5 months old, was positive by MHT (1/6). All congenital CD cases were positive by PCR tests (10/10), and *Tcruzi*-LAMP yielded a positive result in 9 of them (9/10). The undetected case was a child older than 9 months of age (case 38; Table 2). For uninfected children, *Tcruzi*-LAMP and kDNA-PCR yielded negative results in all of them (23/23), whereas Sat-qPCR returned a positive result in 1 infant (Table S1 summarizes the results that rule out T. cruzi infection in uninfected children).

**FIG 3:**
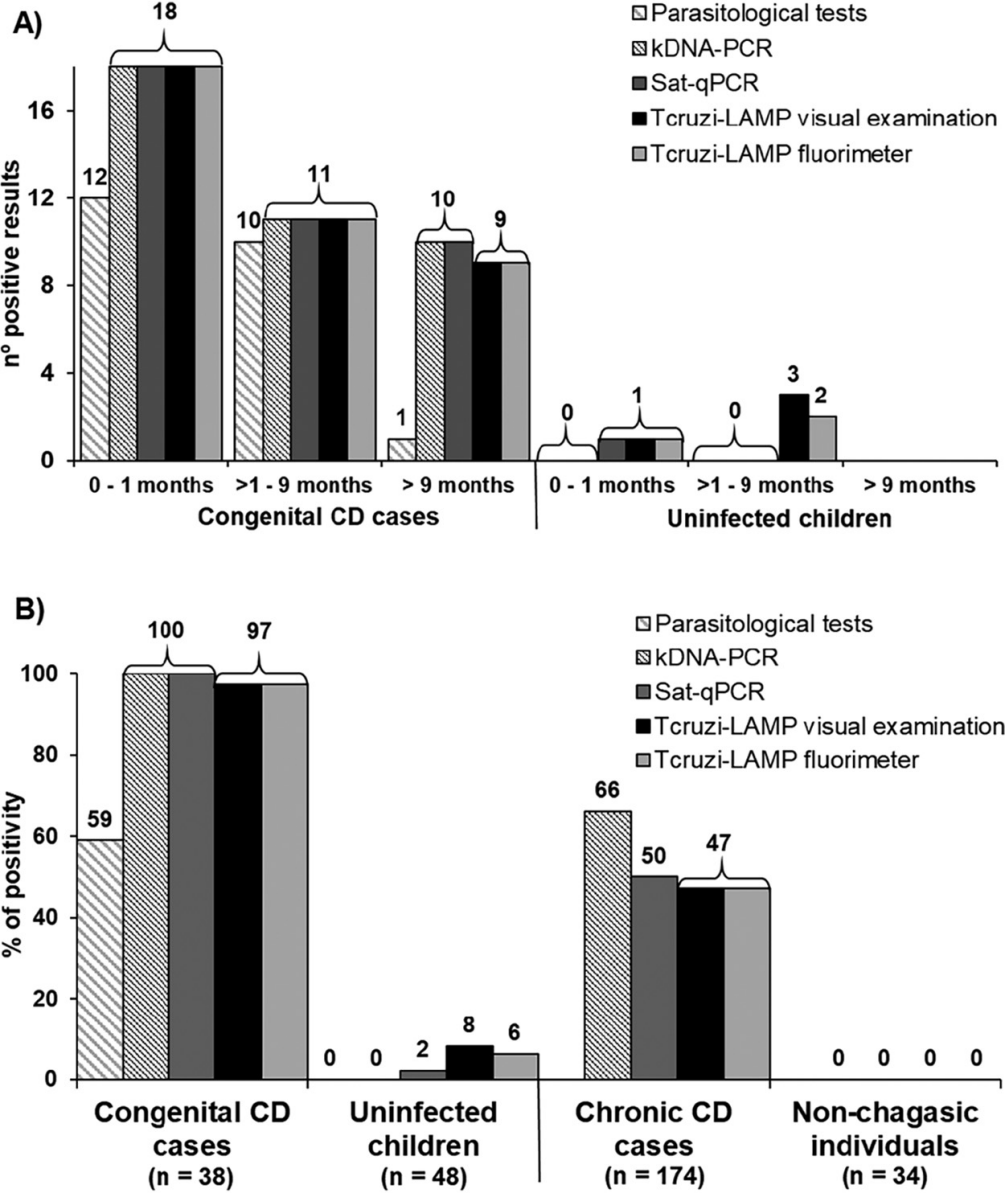
Positivity rate of parasitological tests, *Tcruzi*-LAMP, and PCR tests. (A) Children born in Spain to mothers with T. cruzi infection. (B) Comparison between sample groups. In congenital CD cases, the parasitological tests were performed in 28 samples and 23 were positive; to calculate the percentage of positivity, numerators were positive cases by each test and denominators were total cases.

When comparing the global positivity of parasitological and molecular tests (Fig. 3A) among 12 infected children diagnosed from 0 to 1 month after birth, 83.3% (10/12) had circulating parasites in the bloodstream. From 1 to 9 months after birth, 92.3% (12/13) of cases were positive by parasitological tests. For infants older than 9 months of age, only 1 (1/3) was positive for parasite detection. Both *Tcruzi*-LAMP and PCR tests detected 100% (29/29) of infected children younger than 9 months of age. Among infants older than 9 months found positive by serological and PCR tests (10/10), the 1 case not detected by *Tcruzi*-LAMP (1/10) had a low degree of parasitemia, while most of the infected children (25/39) showed levels of parasitemias higher than 40 parasites/ml (Tables 3 and 5).

**TABLE 3:** Time to positivity by *Tcruzi*-LAMP and degree of parasitemia estimated by Sat-qPCR according to clinical status

Test and result stratification	Congenital CD cases (*n* = 39)		Chronic CD cases (*n* = 174)	
	No.	%	No.	%
*Tcruzi*-LAMP fluorimeter	38	97.4	82	47.1
Time to positivity^*a*^
10:00 to 20:00	34	89.5	49	59.8
21:00 to 30:00	4	10.5	25	30.5
31:00 to 40:00	0	0	8	9.8
Sat-qPCR	39	100.0	87	50.0
Degree of parasitemia^*a*^
Not quantifiable	0	0	19	21.8
<40 par/ml	14	35.9	44	50.6
41–10,000 par/ml	17	43.6	23	26.4
>10,000 par/ml	8	20.5	1	1.1

a Differences in time of positivity and degree of parasitemia between congenital CD versus chronic CD were determined using the Mann-Whitney U test (for both comparisons, *P* < 0.001). For analysis, the categorical factor was infection (congenital infection, chronic infection), and the continuous responses were time to positivity and degree of parasitemia. Degree of parasitemia ranges were determined considering the limit of detection (LOD) of a microhematocrit test according to Vera-Ku et al. (LOD, 10,000 par/ml) (33) and according to Torrico et al. (LOD, 40 par/ml) (26). Par, parasites.

In summary, T. cruzi parasite detection (82.1%, 23/28) was lower than T. cruzi DNA detection (97.4%, 38/39 by *Tcruzi*-LAMP; 100%, 39/39 by PCR tests). In *Tcruzi*-LAMP, 89.5% (34/39) returned as positive in under 20 min. In 64.1% of congenital cases, the degree of parasitemia was above 40 parasites per ml (Table 3).

### Chronic Chagas disease.

For chronic CD cases, 82 (47.1%) of the samples were positive by *Tcruzi*-LAMP, 115 (66.1%) by kDNA-PCR, and 87 (50%) by Sat-qPCR. None of the molecular tests were positive in nonchagasic individuals (Fig. 3). In *Tcruzi*-LAMP, no differences between the fluorimeter and visual examination readings were observed.

Unlike what is observed in congenital infection, *Tcruzi*-LAMP returned a positive result before 20 min, just in 59.8% of chronic CD cases, and in 78.1% of cases, the level of parasitemia was lower than 40 parasites/ml (Table 3; *P* < 0.001).

### Sensitivity, specificity, and accuracy.

*Tcruzi*-LAMP for congenital CD diagnosis displayed a sensitivity of 97.4% for both types of readings and 100% for infected children younger than 9 months of age (Table 4), while, depending on the type of reading, specificity was 91.7% by visual examination and 93.8% by fluorimeter. Subsequently, the accuracy for both types of reading and situations was similar, between 94.2% and 96.1%. Differences in performance of *Tcruzi*-LAMP were not statistically significant (*P* > 0.05).

**TABLE 4:** Performance of *Tcruzi*-LAMP estimated by binomial analysis^*a*^

Test	Infection status		Sensitivity			Specificity			Accuracy		
	Positive	Negative	%	95% CI		%	95% CI		%	95% CI	
In congenital infection	
All children
*Tcruzi*-LAMP visual examination
Positive	38	4	97.4	86.5	99.9	91.7	80.0	97.7	94.2	87.1	98.1
Negative	1	44
*Tcruzi*-LAMP fluorimeter
Positive	38	3	97.4	86.5	99.9	93.8	82.8	98.7	95.4	88.6	98.7
Negative	1	45
Children younger than 9 months of age
*Tcruzi*-LAMP visual examination
Positive	29	4	100	88.1	100	91.5	80.0	97.6	94.8	87.2	98.6
Negative	0	43
*Tcruzi*-LAMP fluorimeter
Positive	29	3	100	88.1	100	93.6	82.4	98.7	96.1	89.0	99.2
Negative	0	44
In chronic infection	
*Tcruzi-*LAMP visual examination
Positive	82	0	47.1	39.5	54.8	100	93.4	100	55.8	48.7	62.6
Negative	92	34
*Tcruzi-*LAMP fluorimeter
Positive	82	0	47.1	39.5	54.8	100	93.4	100	55.8	48.7	62.6
Negative	92	34

a According to McNemar’s test, the differences for both types of reading in congenital infection were not statistically significant (*P* > 0.05).

In chronic CD diagnosis, sensitivity for *Tcruzi*-LAMP was low (47.1%), but specificity was 100%. As a consequence, accuracy was 55.8%. Performance of *Tcruzi*-LAMP was similar to that of Sat-qPCR (*P* = 0.332) but different from that of kDNA-PCR (*P* < 0.001).

### Agreement analysis.

The frequency of concordant results between molecular tests was high for congenital CD diagnosis, 97.4% in positive cases and 89.6% in uninfected children (Table 5); thus, the agreement was almost perfect (κ of 0.89 and 95% confidence interval [CI] of 0.79 to 0.98, to κ of 0.91 and 95% CI of 0.82 to 1.00; Table S3).

**TABLE 5:** Observed frequency of the agreement profiles and their relationship with degree of parasitemia^*a*^

Category	*Tcruzi-*LAMP		kDNA-PCR	Sat-qPCR	Observed frequency (%)	Degree of parasitemia (parasites/ml)		
	Visual examination	Fluorimeter				Median	Min	Max
Congenital CD cases (*n* = 39)	+	+	+	+	38 (97.4)^*b*^	917	0.3	1.1E5
	−	-	+	+	1 (2.6)^*c*^	0.1	0.1	0.1
Chronic CD cases (*n* = 174)	+	+	+	+	76 (43.7)^*b*^	5	<0.01^*d*^	1.1E4
	+	+	+	−	5 (2.9)^*c*^
	+	+	−	−	1 (0.6)^*c*^
	−	−	+	+	11 (6.3)^*c*^	0.3	<0.01^*d*^	32.9
	−	−	+	−	23 (13.2)^*c*^
	−	−	−	−	58 (33.3)
Uninfected children (*n* = 48)	+	-	−	−	1 (2.1)
	+	+	−	−	3 (6.2)
	−	−	−	+	1 (2.1)	0.01	0.01	0.01
	−	−	−	−	43 (89.6)
Nonchagasic individuals (*n* = 34)	−	−	−	−	34 (100)

a +, positive result; −, negative result; min, minimum; max, maximum.

b Concordant group.

c Discordant group.

d <0.01, not-quantifiable value. For analysis, not-quantifiable values were considered equivalent to the limit of detection. Differences in quantifiable parasitemia between concordant and discordant groups were determined using the Mann-Whitney U test (*P* < 0.001). For this analysis, the categorical factor was concordance (concordant group, discordant group), and the continuous response was degree of parasitemia.

For chronic CD diagnosis, the frequency of concordant results in nonchagasic individuals was 100%, whereas in chagasic cases it was 77% (κ of  0.67 and 95% CI of 0.58 to 0.77, to κ of 0.83 and 95% CI of 0.75 to 0.91, substantial agreement; Table S3).

Out of 155 samples from congenital and chronic CD cases with a positive result in any molecular test, 114 were positive for all tests (73.6%; 95% CI, 65.9 to 80.3%), and the disagreement percentages were 22.6% (35/155) for *Tcruzi*-LAMP, 0.7% (1/155) for kDNA-PCR, and 18.7% (29/155) for Sat-qPCR. The disagreement was more frequent in samples with a low degree of parasitemia (*P* < 0.001; Table 5).

Comparing the time to positivity of *Tcruzi*-LAMP with the cycle threshold (*C_T_*) values of Sat-qPCR, the correlation was strong in congenital infection (*r*_s_, 0.653; *P* < 0.001) and moderate in chronic CD (*r*_s_, 0.458; *P* < 0.001).

## DISCUSSION

Our data provide substantial evidence on the usefulness of *Tcruzi*-LAMP and its role as a molecular test in the diagnosis of congenital CD in settings of nonendemicity. As the prevalence of congenital T. cruzi infection in countries of nonendemicity is quite low, our results are relevant since we include a cohort with a high number of cases (*n* = 39).

According to the Pan American Health Organization and the World Health Organization, direct parasitological techniques (MHT and direct observation) remain the gold standard for parasite detection in the diagnosis of congenital CD. Should these tests be negative, or if no tests were done before, detection of T. cruzi-specific antibodies in infants from 8 to 10 months old becomes the gold standard (31, 32).

Although MHT is the fastest method to detect T. cruzi (less than 20 min), its sensitivity depends on the operator’s skills, the degree of parasitemia, and whether the sample is examined within 12 h after collection (13, 26, 33). Reports from some South American countries showed that MHT sensitivity was 34.2% in the first month of age (34), 76.1% up to 6 months of age (15), and 94% in 1 year of follow-up (14), whereas qPCR could detect up to 84.2% of infected infants before 1 month of age (34). Others, using conventional PCR, achieved 100% sensitivity in infected children who were diagnosed before 6 months of age (35). Due to losses to follow-up, there is not enough PCR data for the first year of life in children born to T. cruzi-infected mothers.

In Spain, both parasitological tests and PCR are used without distinction for congenital CD diagnosis. Recently, Basile et al. reported that among 22 congenital CD cases born in Catalonia, in 1 year of follow-up, 5 were diagnosed by MHT and 10 by qPCR (36), while in Murcia, among 12 infected infants, 1 was positive by MHT, 8 by culture, and 100% by conventional PCR, also in 1 year of follow-up (18).

There is less information on the use of LAMP for CD diagnosis. So far, three studies were published in populations in areas of endemicity. The first targeted the 18S rRNA gene (37), and the other two, a prototype, targeted SatDNA (21, 38). The first two showed the potential of LAMP in a limited number of samples (seven and five cases, respectively), while the third study showed good sensitivity detecting 10 cases of congenital CD.

In our study, *Tcruzi*-LAMP and PCR tests detected 100% (29/29) of cases younger than 9 months, while parasitological tests detected just 88% (22/25) of them (Table 1). In infants older than 9 months, *Tcruzi*-LAMP was negative in one case (1/10) on a stored DNA sample that could have degraded even under the best storage conditions, considering that this sample was collected in February 2008 and stored at −40°C. This sample was positive by kDNA-PCR and Sat-qPCR tested at the same time. This result may reflect a slightly lower analytical sensitivity for *Tcruzi-*LAMP. Certainly, it is well known that the number of copies of SatDNA in TcI, TcIII, and TcIV is lower than that in TcII, TcV, and TcVI (24, 25, 29, 39). Thus, a low degree of parasitemia could be the cause of a lower sensitivity of techniques targeting SatDNA, such as *Tcruzi*-LAMP or Sat-qPCR (40).

Regarding specificity, four uninfected infants were found positive by *Tcruzi-*LAMP and one by Sat-qPCR, yet all had negative kDNA-PCR and serology after 9 months of age (Table S1). This may be explained by contamination at any step of the procedure. False positives due to contamination can happen in any laboratory, even when using automated systems (34), but this is easy to trace and control with good laboratory practices (41). For this study, new DNA extractions and *Tcruzi*-LAMP were performed by two new laboratory technicians, and the controls (tested in parallel) yielded the expected results. In our experience, the best way to detect false positives due to contamination is to examine the sample in duplicate after DNA extraction and to retest when a positive result is observed in the later steps of amplification. Due to the scarcity of available biological material, we were not able to repeat the DNA extraction to confirm whether these false-positive results were due to contamination during that process. In experiments not shown, it was observed that guanidine produced a color change similar to that observed in a positive sample, but this change was not detected by the fluorimeter. That is, if the guanidine had not been correctly eliminated during DNA extraction, we will have false positives by visual examination but not by fluorimeter. In our study, one of the false positives could have been caused by the presence of guanidine traces. In the other three samples, we cannot discard nonspecific amplification, though.

In terms of cost, the implementation of *Tcruzi*-LAMP could be less expensive than PCR (31). Comparing ready-to-use techniques, *Tcruzi*-LAMP is the only low-cost test (*Tcruzi*-LAMP €7.5 versus commercial qPCR €20 per reaction) giving results faster than the current qPCRs and that can be performed without needing sophisticated instrumentation. The additional advantage of using a real-time isothermal fluorimeter is that the reading eliminates any subjectivity, and time to positivity may allow for estimating the degree of parasitemia. The correlation between the time to positivity in *Tcruzi*-LAMP and the *C_T_* values of qPCRs was significant in samples from congenital CD, but further research on this observation is required.

The main limitation of our study is the relatively low number of samples of uninfected individuals to estimate specificity. As *Tcruzi*-LAMP is based on SatDNA, we expected low sensitivity; therefore, in the study design, we tended to include a high number of positive cases. In experiments not shown, we tested samples from malaria and leishmaniasis patients with all molecular T. cruzi tests (*n* = 20), and all samples tested negative. Considering these data and results of all uninfected individuals (48 samples from uninfected children and 34 samples from nonchagasic individuals), the overall specificity for *Tcruzi*-LAMP by visual examination was 96.1% (95% CI, 90.3 to 98.9%) and by fluorimeter was 97.1% (95% CI, 91.6 to 99.4%). New studies will improve these estimates.

Although this study was retrospective and based on convenience and available sampling, the results clearly support the usefulness of the Loopamp Trypanosoma cruzi detection kit in the early diagnosis of congenital T. cruzi infection. *Tcruzi*-LAMP showed a suitable performance, and running in duplicate, it could be used as a screening test before 9 months of age and afterward as an alternative or complement to current diagnostic tools. In the chronic phase, its role would be similar to that of the Sat-qPCR.

## Supplementary Material

Supplemental file 1

Supplemental file 2

Supplemental file 3
