# Supplementary material for: Evaluation of the Performance of the Loopamp Trypanosoma cruzi Detection Kit for the Diagnosis of Chagas Disease in an Area Where It Is Not Endemic, Spain
Source: J Clin Microbiol. 2021 Apr 20;59(5):e01860-20. doi: 10.1128/JCM.01860-20 (PMC8091841; doi:10.1128/JCM.01860-20)
Supplement: Supplemental file 1 [file JCM.01860-20-s0001.pdf]

SUPPLEMENTAL MATERIAL

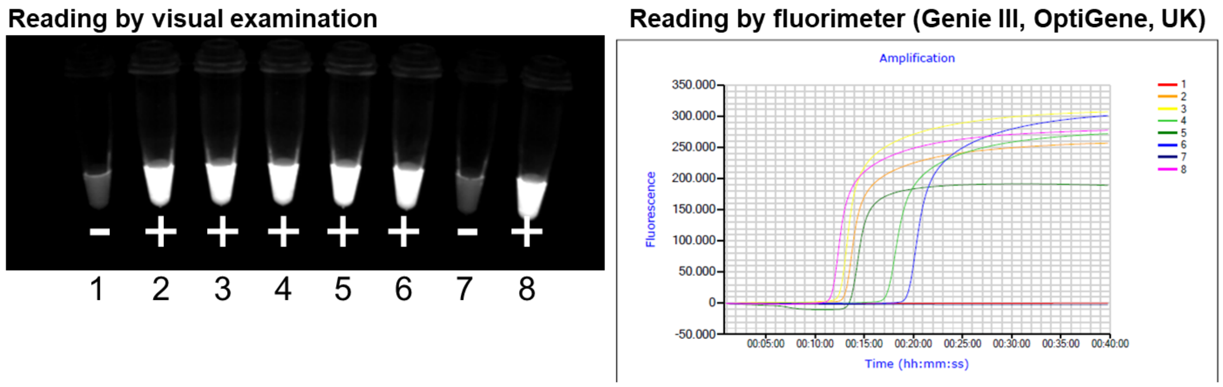

Well Name	Peak Value(mm:ss)
2	13:45
3	13:15
4	18:00
5	14:15
6	20:15
8	12:15

FIGURE S1. Example a run of Loopamp™ *Trypanosoma cruzi* Detection Kit LAMP (Index test). 1 – 6 samples, 7 negative control, 8 positive control. Patient samples were tested once and in a single reaction. Five microliters of DNA purified by Roche columns and 25 µL of free DNAse water were deposited at the bottom of the microtube. The cap containing the lyophilized LAMP reagents was then closed. One positive control and one negative control were processed in each microtube strip (eight reactions in each strip). After mixing by inversion, the strip of tubes were left standing for 2 min in the dark and centrifuged for 15 seconds. Immediately after this, the microtubes were put in the real time isothermal fluorimeter (Genie® III, OptiGene, UK). Amplification conditions were a first step of 5 min at 95 ºC followed by 40 min at 65 ºC. The naked eye reading was done immediately after the end of the reaction; no color change was recorded as a negative result, and a color change as a positive result. The results were read by two operators who were blinded to previous results. The fluorimeter reading was displayed at the end of the reaction as time to positivity in minutes and seconds. The results of each run were validated when positive and negative controls yielded expected results.

TABLE S1. Characteristic at baseline of samples from at-risk children born in Spain to *T. cruzi* infected mothers

	**Samples from CNM**		**Samples from PFSF-UB/ HSCSP**	
**CONGENITAL CD CASES**				
**Gender (n, %)**				
Female	13	44.8	5	50
Male	16	55.2	5	50
**Age**				
0 -1 months	11	37.9	5	50
1 - < 9 months	11	37.9	2	20
> 9 months	7	24.1	3	30
**Parasitological tests**				
Positive	22	75.9	1	10
Negative	0	0	5	50
Not done	7	24.1	4	40
**kDNA-PCR**				
Positive	29	100	0	0
Not done	0	0	10	100
**Sat-qPCR**				
Positive	0	0	10	100
Not done	29	100	0	0
**Serological tests**				
Positive	29	100	7	70
Not done	0	0	3	30
**UNINFECTED CHILDREN**				
**Gender (n, %)**				
Female	8	32	5	21.7
Male	17	68	18	78.3
**Age**				
0 -1 months	11	44	23	100
1 - < 9 months	13	52	0	0
> 9 months	1	4	0	0
**Parasitological tests**				
Negative	21	84	14	60.9
Not done	4	16	9	39.1
**kDNA-PCR**				
Negative	25	100	0	0
Not done	0	0	23	100
**Serological tests at time of diagnosis**				
Positive	21	84	13	56.5
Negative	4	16	0	0
Not done	0	0	10	43.5
**Serological tests at after 9 months**				
Negative	25	100	23	100

These samples were included considering the available volume to carry out new DNA extraction by Roche columns for CNM samples or all molecular tests for PFSF-UB/ HSCSP samples.

TABLE S2. Characteristic at baseline of sample from at-risk older children/adults for chronic *T. cruzi* infection

	Infected (n = 174)		Uninfected (n = 34)	
**Gender (n, %)**				
Female	111	63.8	20	58.8
Male	63	36.2	14	41.2
**Age (mean, range)**	39.1	10-65	38.9	11-65
**Country of Origin (n, %)**				
Bolivia	128	73.6	7	20.6
Brazil	2	1.1	1	2.9
Paraguay	1	0.6	0	0
Other endemic countries*	43	24.7	1	2.9
Argentina	0	0	1	2.9
Cape Verde**	0	0	1	2.9
Colombia	0	0	1	2.9
Peru	0	0	1	2.9
Spain**	0	0	21	61.8
**Serological tests (n, %)**				
Positive	174	100	0	0
Negative	0	0	34	100
**kDNA-PCR (n, %)**				
Positive	115	66.1	0	0
Negative	59	33.9	34	100

*Specific country was not recorded. **People with a history of long staying or past travels to endemic countries.

TABLE S3. Agreement between molecular tests by Cohen's kappa coefficient and 95% confidence intervals

	***Tcruzi*-LAMP visual examination**	***Tcruzi*-LAMP fluorimeter**	**kDNA-PCR**	**Sat-qPCR**
**CONGENITAL INFECTION**				
Tcruzi-LAMP visual examination	1.00<br>(1.00-1.00)			
Tcruzi-LAMP fluorimeter	0.98<br>(0.93-1.00)	1.00<br>(1.00-1.00)		
kDNA-PCR	0.89<br>(0.79-0.98)	0.91<br>(0.82-1.00)	1.00<br>(1.00-1.00)	
Sat-qPCR	0.86<br>(0.76-0.97)	0.89<br>(0.79-0.98)	0.98<br>(0.93-1.00)	1.00<br>(1.00-1.00)
**CHRONIC INFECTION**				
Tcruzi-LAMP visual examination	1.00<br>(1.00-1.00)			
Tcruzi-LAMP fluorimeter	1.00<br>(1.00-1.00)	1.00<br>(1.00-1.00)		
kDNA-PCR	0.67<br>(0.58-0.77)	0.67<br>(0.58-0.77)	1.00<br>(1.00-1.00)	
Sat-qPCR	0.83<br>(0.75-0.91)	0.83<br>(0.75-0.91)	0.74<br>(0.65-0.82)	1.00<br>(1.00-1.00)
