# Supplementary material for: Evaluation of the Performance of the Loopamp Trypanosoma cruzi Detection Kit for the Diagnosis of Chagas Disease in an Area Where It Is Not Endemic, Spain
Source: J Clin Microbiol. 2021 Apr 20;59(5):e01860-20. doi: 10.1128/JCM.01860-20 (PMC8091841; doi:10.1128/JCM.01860-20)
Supplement: Supplemental file 2 [file JCM.01860-20-s0002.pdf]

Section & Topic	No	Item	Reported on page #
**TITLE OR ABSTRACT**			
	**1**	Identification as a study of diagnostic accuracy using at least one measure of accuracy<br>(such as sensitivity, specificity, predictive values, or AUC)	1-2
**ABSTRACT**			
	**2**	Structured summary of study design, methods, results, and conclusions<br>(for specific guidance, see STARD for Abstracts)	2
**INTRODUCTION**			
	**3**	Scientific and clinical background, including the intended use and clinical role of the index test	3-4
	**4**	Study objectives and hypotheses	4
**METHODS**			
Study design	**5**	Whether data collection was planned before the index test and reference standard<br>were performed (prospective study) or after (retrospective study)	4
Participants	**6**	Eligibility criteria	4
	**7**	On what basis potentially eligible participants were identified<br>(such as symptoms, results from previous tests, inclusion in registry)	5
	**8**	Where and when potentially eligible participants were identified<br>(setting, location and dates)	5, Table S1
	**9**	Whether participants formed a consecutive, random or convenience series	4
Test methods	**10a**	Index test, in sufficient detail to allow replication	6, Fig S4
	**10b**	Reference standard, in sufficient detail to allow replication	7-8
	**11**	Rationale for choosing the reference standard (if alternatives exist)	6
	**12a**	Definition of and rationale for test positivity cut-offs or result categories<br>of the index test, distinguishing pre-specified from exploratory	6, Fig S4
	**12b**	Definition of and rationale for test positivity cut-offs or result categories<br>of the reference standard, distinguishing pre-specified from exploratory	5
	**13a**	Whether clinical information and reference standard results were available<br>to the performers/readers of the index test	6
	**13b**	Whether clinical information and index test results were available<br>to the assessors of the reference standard	n.a
Analysis	**14**	Methods for estimating or comparing measures of diagnostic accuracy	7-8
	**15**	How indeterminate index test or reference standard results were handled	n.a.
	**16**	How missing data on the index test and reference standard were handled	n.a.
	**17**	Any analyses of variability in diagnostic accuracy, distinguishing pre-specified from exploratory	8
	**18**	Intended sample size and how it was determined	n.a.
**RESULTS**			
Participants	**19**	Flow of participants, using a diagram	8, 27-28

	**20**	Baseline demographic and clinical characteristics of participants	9, 22, Table S2 and S3
	**21a**	Distribution of severity of disease in those with the target condition	22
	**21b**	Distribution of alternative diagnoses in those without the target condition	Table S2
	**22**	Time interval and any clinical interventions between index test and reference standard	n.a.
Test results	**23**	Cross tabulation of the index test results (or their distribution) by the results of the reference standard	24 (Table 3)
	**24**	Estimates of diagnostic accuracy and their precision (such as 95% confidence intervals)	24 (Table 3)
	**25**	Any adverse events from performing the index test or the reference standard	9, 23 (Table 2)
**DISCUSSION**			
	**26**	Study limitations, including sources of potential bias, statistical uncertainty, and generalisability	11-13
	**27**	Implications for practice, including the intended use and clinical role of the index test	11-13
**OTHER INFORMATION**			
	**28**	Registration number and name of registry	8, 13
	**29**	Where the full study protocol can be accessed	4-8
	**30**	Sources of funding and other support; role of funders	13

n.a., not applicable. S.A., Supplementary Appendix.
